# Far- and deep-ultraviolet surface plasmon resonance sensors working in aqueous solutions using aluminum thin films

**DOI:** 10.1038/s41598-017-06403-9

**Published:** 2017-07-19

**Authors:** Ichiro Tanabe, Yoshito Y. Tanaka, Koji Watari, Taras Hanulia, Takeyoshi Goto, Wataru Inami, Yoshimasa Kawata, Yukihiro Ozaki

**Affiliations:** 1 Graduate School of Engineering Science, Osaka University, Machikaneyama 1-3, Toyonaka, Osaka Japan; 2 Institute of Industrial Science, the University of Tokyo, Komaba 4-6-1, Meguro, Tokyo Japan; 3 School of Science and Technology, Kwansei Gakuin University, Gakuen 2-1, Sanda, Hyogo Japan; 4 Research Institute of Electronics, Shizuoka University, Johoku 3-5-1, Hamamatsu, Shizuoka Japan

## Abstract

Surface plasmon resonance (SPR) sensors detect refractive index changes on metal thin films and are frequently used in aqueous solutions as bio- and chemical-sensors. Recently, we proposed new SPR sensors using aluminum (Al) thin films that work in the far- and deep-ultraviolet (FUV-DUV, 120–300 nm) regions and investigated SPR properties by an attenuated total reflectance (ATR) based spectrometer. The FUV-DUV-SPR sensors are expected to have three advantages compared to visible-SPR sensors: higher sensitivity, material selectivity, and surface specificity. However, in this study, it was revealed that the Al thin film on a quartz prism cannot be used as the FUV-DUV-SPR sensor in water solutions. This is because its SPR wavelength shifts to the visible region owing to the presence of water. On the other hand, the SPR wavelength of the Al thin film on the sapphire prism remained in the DUV region even in water. In addition, the SPR wavelength shifted to longer wavelengths with increasing refractive index on the Al thin film. These results mean that the Al thin film on the sapphire prism can be used as the FUV-DUV-SPR sensor in solutions, which may lead to the development of novel and sophisticated SPR sensors.

## Introduction

Surface plasmon resonance (SPR) sensors detect refractive index changes near the surface of a metal thin film as changes in the intensity of reflected light, which are induced by SPR wavelength and angle shifts. SPR sensors have been used in various fields such as medical diagnostics^1–3^ and environmental monitoring^4, 5^. For example, SPR sensors are widely used in biosensors for medical diagnostics, and the reported best Limit of Detection (LOD) of cancer markers is less than 1 ng mL^−1^ ^3^. In practical applications, changes in the intensity of reflected light are detected at a fixed angle and wavelength. To determine the suitable wavelength for SPR sensors, it is necessary to obtain the reflection spectra.

Recently, we proposed new SPR sensors that use short-wavelength light in the deep-ultraviolet (DUV, 200‒300 nm) and the far‒ultraviolet (FUV, 120‒200 nm) regions, while conventional SPR sensors use light in the visible region. SPR and SPR-like systems working in the UV region have been reported by some groups^6–8^. Al is a suitable metal for FUV‒DUV‒SPR studies because its plasma frequency (2.4 × 10^16^ s^−1^)^9^ is higher than the light frequency in the FUV and DUV regions. The plasma frequencies of Au and Ag are 1.37 × 10^16^ s^−1^ and 1.36 × 10^16^ s^−1^, respectively^10, 11^, and Au and Ag thin films are widely utilized for visible-SPR sensors. Although many target molecules of SPR sensors such as DNA and proteins have no absorbance in the visible region, they have strong absorption and high refractive indices in the FUV-DUV region^12, 13^. In addition, the penetration depth of the evanescent wave in the FUV-DUV region is less than 50 nm, which is much shorter than that in the visible region. Owing to the higher refractive indices of target molecules and the shorter penetration depth of the evanescent light, FUV-DUV-SPR sensors are expected to have three remarkable properties: high sensitivity, material selectivity, and surface measurement accuracy; these properties were discussed in our previous paper^14^. For example, we demonstrated that the shift in the SPR angle caused by the presence of 1,1,1,3,3,3-hexafluoro-2-propanol (HFIP) for the FUV-DUV-SPR sensor was larger than that for the visible-SPR sensor^14^. In addition, according to simulations based on the Fresnel equations, FUV-DUV-SPR sensors can detect refractive index changes in the 2-nm-thick region on the metal film 16 times more sensitively than visible-SPR sensors^14^.

However, until quite recently, investigations of the SPR properties in the FUV regions were done only in a high vacuum atmosphere^15–17^, and there was no report about FUV-DUV-SPR sensors. This is because atmospheric H_2_O and O_2_ molecules yield very intense absorptions in the FUV region, so the environment on the metal surface could not be controlled. Therefore, the measurement ranges in most Al-SPR studies were limited to the DUV region (>200 nm)^18–23^. In contrast, we have recently reported the FUV-DUV-SPR properties of Al thin films with varying surface refractive indices by putting an organic liquid on the Al films^14^. To obtain the reflection spectra in the FUV region, a new attenuated total reflectance (ATR) spectrometer was developed. In this spectrometer, the optics part and the sample part are separated by an internal reflection element (IRE)^24–27^. The sample part is exposed to air, and the environment around the sample can be controlled. Using this ATR system, we measured the SPR properties of the Al thin film on a quartz prism in the 180–300 nm wavelength region and the 45°–80° angle region^14^. By increasing the refractive index *n* on the Al film, the SPR angle and SPR wavelength became larger and longer, respectively, compared to those in the air.

In this study, the SPR wavelength was measured for Al thin films evaporated on quartz and sapphire prisms by varying the refractive indices on the Al films. On replacing the quartz prism with a sapphire prism, the SPR wavelength became shorter, from 334.4 nm to 215.2 nm in HFIP. The SPR wavelength of the Al thin film on the sapphire prism was still in the DUV region (~227.5 nm), even in water. As conventional SPR sensors are often used in aqueous solutions as biosensors and chemical sensors, it is expected that the FUV-DUV-SPR sensors will also be used aqueous solutions. The present Al thin film on the sapphire prism showed the red-shift of the SPR wavelength in the DUV region on increasing the concentration of the sucrose water solution, which may lead to the development of the FUV-DUV-SPR sensor working in the solutions.

## Experimental

The Kretschmann configuration was used to excite the SPR of an Al film deposited on quartz and sapphire prisms (purchased from Opto-line, Tokyo) by vapours deposition (5.0 × 10^−4^ Pa, deposition rate ~10 nm s^−1^). Their thickness and surface roughness, measured by atomic force microscopy, were approximately 23.0 ± 0.5 nm.

The prisms were set on the ATR spectrometer, and the reflection spectra were obtained with the incident angle at 70°. A deuterium lamp and a halogen lamp were used as the light sources for the spectral measurements in the 170–450 nm and 400–650 nm regions, respectively. The optics section is purged with dry N_2_ gas, which does not absorb FUV light. The details of the system are described in the previous paper^14^.

In order to change the refractive index of the environment near the evaporated Al film, HFIP and water were cast on the Al film. Subsequently, sucrose water solutions with various concentrations (0.025–0.250 M) were cast on the Al film to increase the refractive index little by little. It should be noted here that these liquids have no significant absorbance in the measured wavelength region.

In order to analyse the experimentally obtained reflection spectra, reflection spectra were simulated according to the Fresnel equations based on a bilayer (aluminium and alumina) model with varying refractive index of the environment on the film.

## Results and Discussion

### SPR properties of the Al film on the quartz prism

**Figure 1:**
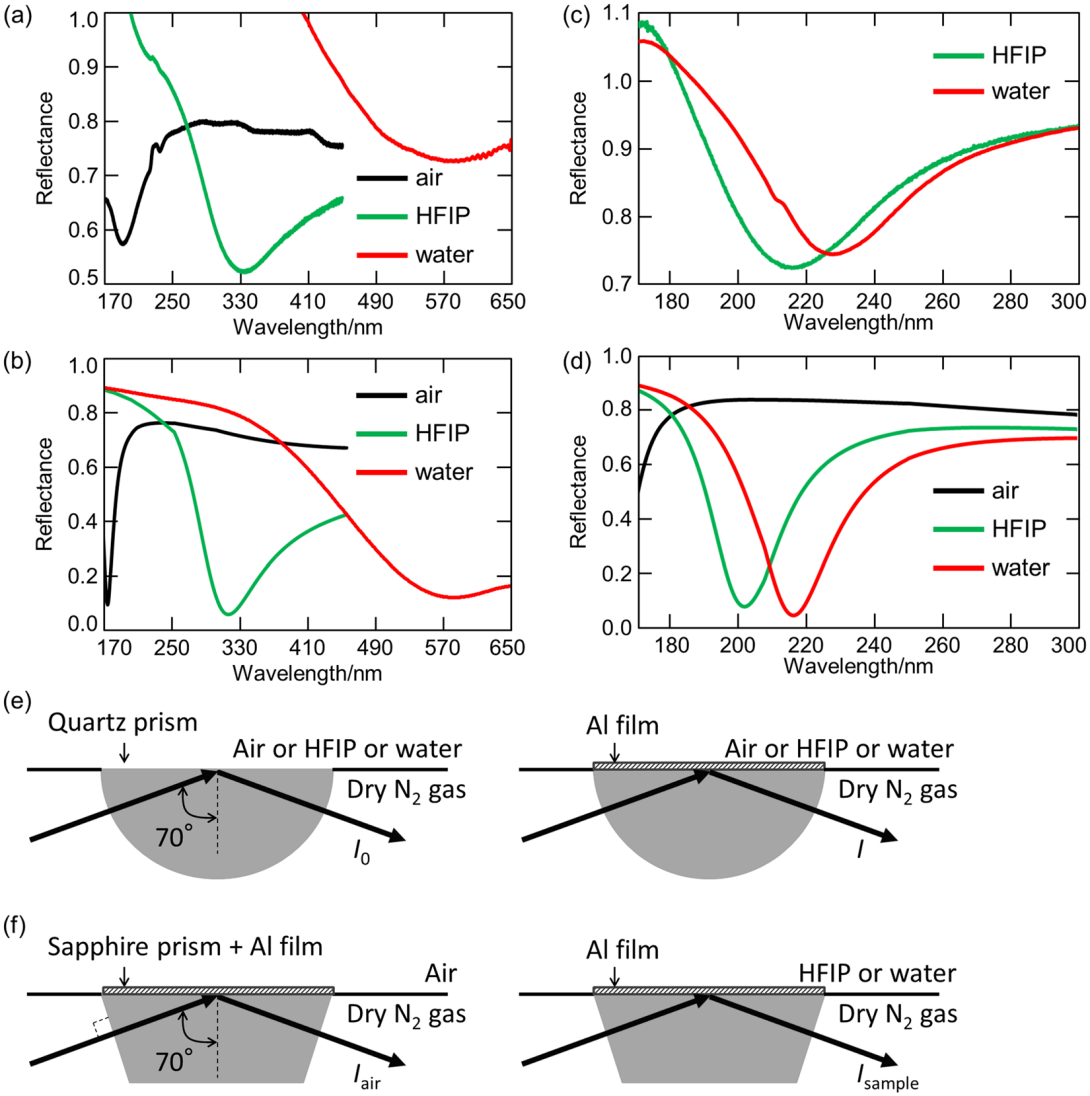
(**a**,**c**) Experimental and (**b**,**d**) simulated reflection spectra and (**e**,**f**) experimental schematics of the Al thin film on (**a**,**b**,**e**) the quartz prism and (**c**,**d**,**f**) the sapphire prism in (black) air, (green) HFIP, and (red) water.

Figure 1a shows the reflection spectra in the 170–650 nm region of the Al thin film on the quartz prism in air, HFIP, and water. In the case of the Al thin film on the semi-cylindrical quartz prism, the reflection spectra of a quartz prism without Al film were measured and used as reference. Reflectance is defined as *I*/*I*_0_, where *I* and *I*_0_ are the reflected light intensities from the quartz prism with and without Al film, as shown in Fig. 1e, respectively. Spectral dips were observed at ~190.3 nm in air (*n* = 1.000), ~334.4 nm in the HFIP (n_D_ = 1.275), and ~568.0 nm in water (n_D_ = 1.333), respectively, which means that the Al-SPR was excited at these wavelengths. The value of n_D_ represents the refractive index at 589.3 nm, which is the wavelength of the sodium D line. Although only *p*-polarized light can excite the SPR, the present incident light includes both *p*-polarized and *s*-polarized light. As a result, the minimum reflectance value is around 0.5 at most. Because of the space limitation of the present system, it is difficult to set a polarizer for the FUV-DUV region. When the reflection spectra were measured by the fourth harmonic of a Nf:YAG laser (266 nm), reflectance at the SPR angle became almost 0 because of *p*-polarized light^14^.

It should be noted here that Al is easily oxidized, and oxidation has significant effects on SPR properties^27, 28^. Our previous work revealed that the reflection spectra of the prepared film can be reproduced based on a bilayer (aluminum and alumina) model using the Fresnel equations^14^. When the thicknesses of Al and Al_2_O_3_ are assumed to be 19 nm and 4 nm, respectively, the calculated results show a good agreement with the experimental results. More details are available in our previous paper^14^.

The obtained reflection spectra (Fig. 1a) were simulated by the Fresnel equations using the optimized bilayer (Al 19 nm/Al_2_O_3_ 4 nm) model as shown in Fig. 1b. When the refractive indices *n* on the Al 19 nm/Al_2_O_3_ 4 nm bilayer film were 1.000, 1.275, and 1.333, the calculated SPR wavelengths were 173, 313, and 572 nm, respectively. The calculated wavelengths were shorter than the measured ones because the calculations were done using the refractive indices in the visible region (n_D_). The refractive indices in the short-wavelength region are larger than those in the visible region, which results in larger shifts of the measured SPR wavelength. However, it is difficult for many materials to obtain chromatic dispersion of the refractive indices (i.e. real part *n* and imaginary part *k*) in the FUV and DUV regions because of the huge absorption as described in ref. 22. Considering the refractive index dispersion of HFIP in the FUV-DUV region calculated by ourselves using density functional theory (DFT) in our previous paper^14^, the calculated SPR wavelength shifts to longer wavelengths (from 313 to 331 nm) and approaches the experimentally obtained one (334 nm). As ascribed in Fig. 1e, the prisms were exchanged when *I*_0_ and *I* were measured. Although we tried put the prism on the same position as exactly as possible, the position are different by times, results in the baseline deviations and differences between Fig. 1a and b; reflectance in the short wavelength region in Fig. 1a were over “1.0”. Even so, the SPR wavelengths matched with the simulation results were successfully obtained. It should be also noted that the SPR wavelength in water is in the visible region (~568 nm) when the Al thin film is made on the quartz prism. According to the Fresnel equations, even if the incident angle is larger (89°), the SPR wavelengths in HFIP and water are about 260 nm and 310 nm at most, respectively.

### SPR properties of the Al film on the sapphire prism

Subsequently, Al was evaporated on the trapezoid sapphire prism instead of the quartz prism, and the reflectance spectra in the 170–300 nm region were measured in HFIP and water (Fig. 1c). These liquids were selected because they have little absorbance in the measurement wavelength, which makes it easier to discuss the refractive index dependence of the SPR. In the case of the Al thin film on the sapphire prism, it is difficult to prepare two sapphire prisms that have the same optical properties in the FUV-DUV region. This is because intrinsic defects have significant effects on the optical properties of sapphire^29^, and each sapphire prism has different optical properties. Therefore, differently from the case of a quartz prism, a sapphire prism without Al film cannot be used as reference for another sapphire prism with Al film. Alternatively, reflectance is defined as *I*_sample_/*I*_air_, where *I*_sample_ and *I*_air_ are the reflected light intensities from the Al film measured on a sapphire prism with and without a sample on the Al film, as shown in Fig. 1f, respectively. That is to say, the reflectance spectrum measured in air is used as the reference. According to simulations based on the Fresnel equations, an Al film on a sapphire prism in air has no SPR absorbance and has almost constant reflectance intensity in the 180–300 nm region at a 70° incident angle (Fig. 1d, black line). Thus, in the present study, the reflection spectrum in the air can be used as the reference to determine the SPR wavelength with materials on the Al film.

In Fig. 1c, the SPR wavelengths were observed at ~215.2 nm in HFIP (n_D_ = 1.275) and ~227.5 nm in water (n_D_ = 1.332), respectively. Similarly to the Al thin film on the quartz prism, the SPR wavelength shifts to longer wavelengths with the increment of the refractive index on the Al surface. The experimentally obtained reflection spectra were reproduced by the Fresnel equations using the 4 nm thick Al_2_O_3_ model (Al 19 nm/Al_2_O_3_ 4-nm-bilayer model), as shown in Fig. 1d. The simulated SPR wavelengths were 201 nm and 216 nm when the refractive indices on the Al/Al_2_O_3_ bilayer film were 1.275 and 1.332, respectively.

**Figure 2:**
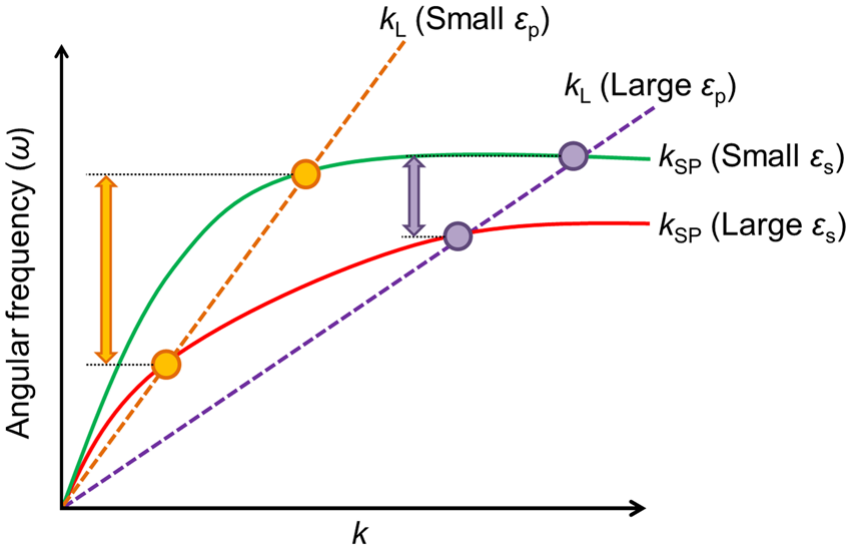
Schematic of the dispersion relations of surface plasmon (solid lines) with small (green) and large (red) surrounding dielectric constants on the metal (*ε*_s_), and the light lines in the prisms (dashed lines) with small (orange) and large (purple) dielectric constants (*ε*_p_). Circles and arrows represent the SPR excitation points and angular frequency differences between the small *ε*_s_ and large *ε*_s_, respectively.

Table 1 summarizes the SPR wavelength and half width at half maximum (HWHM) of the experimentally obtained spectra in Fig. 1a,c. In the presence of HFIP and water on the Al thin films, the SPR wavelength shift of the Al thin film on the quartz prism is larger than that on the sapphire prism. Figure 2 illustrates a schematic of the dispersion relations of the surface plasmon (solid lines) and light lines in the prisms (dashed lines). Intersection points (circles) mean that the surface plasmon is excited at wavenumber *k* and angular frequency *ω*. The surface plasmon dispersion relation and the light line are expressed by equations (1) and (2), where c represents the light speed and *ε*_s_, *ε*_m_, and *ε*_p_ represent the dielectric constants of the surroundings, metals, and prisms, respectively. Theta (*θ*) is the incident angle, and it is 70° in this study.

1 $${k}_{{\rm{S}}{\rm{P}}}=\frac{\omega }{c}\sqrt{\frac{{\varepsilon }_{s}{\varepsilon }_{m}}{{\varepsilon }_{s}+{\varepsilon }_{m}}}$$

2 $${k}_{{\rm{L}}}=\frac{\omega }{c}\sqrt{{\varepsilon }_{p}}\,\sin \,\theta $$

By incrementing *ε*_s_, *ω* in the surface plasmon dispersion relation becomes smaller, which means an alternation from the green line to the red line. When *ε*_p_ increases, the slope of the light line decreases from the orange line to the purple line. As described in Fig. 2, the shift of the resonance angular frequency following an increase in the value of *ε*_s_ in the prism with a smaller *ε*_p_ is larger than that in the prism with a larger *ε*_p_. If a material has no absorbance, its dielectric constant corresponds to the square of its refractive index *n*. The relationship between the angular frequency ω and wavelength *λ* is an inverse proportion. In the present case, by placing materials (*i*.*e*., HFIP and water) on the Al film, the surrounding dielectric constant increases, and the SPR angular frequency becomes smaller (*i*.*e*., red shift of the SPR wavelength). In addition, the refractive index of quartz (~1.5) is smaller than that of sapphire (~1.8); thus, the SPR wavelength shift of the Al on quartz is larger than that on sapphire.

**Table 1:** SPR wavelength and half width at half maximum (HWHM) dependence on the surroundings of the Al thin film on quartz and sapphire prisms.

Prism	Surroundings	SPR wavelength	HWHM
Quartz	Air	190.3 nm	—
Quartz	HFIP	334.4 nm	63 nm
Quartz	Water	568.0 nm	120 nm
Sapphire	HFIP	215.2 nm	25 nm
Sapphire	Water	227.5 nm	30 nm

It should be emphasized that the SPR wavelength of the Al thin film on the sapphire prism exists in the DUV region even in water, which is different from the Al thin film on the quartz prism. Therefore, the Al thin film on the sapphire prism can be used as a FUV-DUV-SPR sensor, which can be expected to have the three advantages previously described, in the water solution. Actually, according to the permittivity of Al and the experimentally obtained SPR wavelength, the penetration depths of the SPR in water are ~120 nm for Al on the quartz prism and ~45 nm for Al on the sapphire prism, respectively. It means that the SPR of the Al thin film on the sapphire prism is sensitive to more narrowly limited surface depth. In addition, as ascribed in Table 1, the Al film on the sapphire prism shows a much narrower HWHM than that on the quartz prism because the SPR wavelength of the Al film on the sapphire prism is in the short-wavelength (i.e., high-energy) region. This is also an advantage of using sapphire prism for SPR sensors.

### SPR wavelength dependence of sucrose concentrations in water

**Figure 3:**
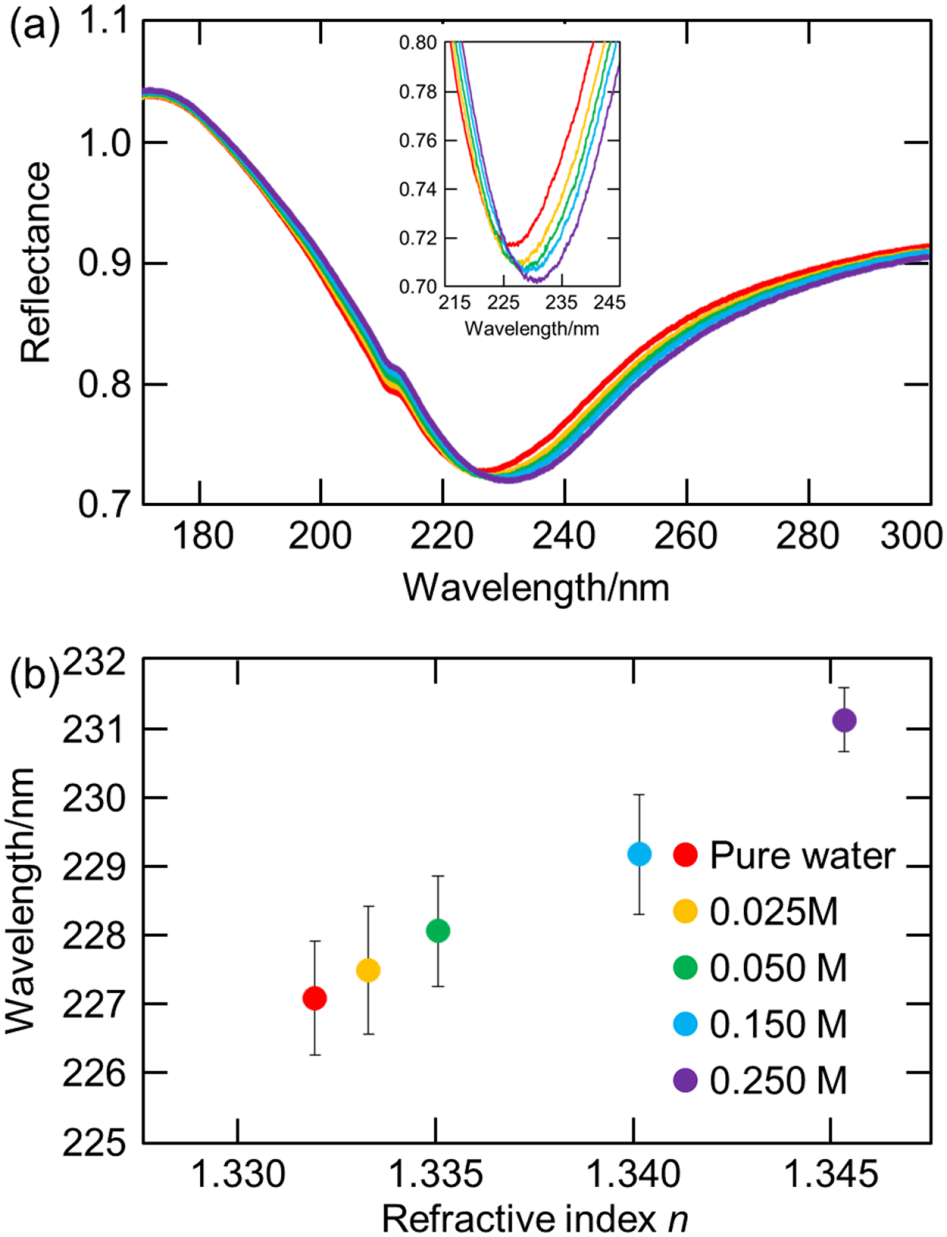
(**a**) Reflection spectra and (**b**) SPR wavelength dependence on the refractive index of the Al thin film on the sapphire prism in pure water and sucrose water solutions with various concentrations (0.025–0.250 M). The refractive index was measured in the visible region.

Figure 3a shows the reflection spectra of the Al thin film on the sapphire prism in pure water and sucrose water solutions with various concentrations (0.025–0.250 M). When the concentrations of the sucrose water solutions were 0 (pure water), 0.025, 0.050, 0.150, and 0.250 M, the refractive indices measured by the refractometer (PAL-RI, Atago Co., Ltd. Japan) were 1.3325, 1.3338, 1.3355, 1.3404, and 1.3454, respectively. The SPR wavelengths were 227.05 ± 0.38, 227.63 ± 0.41, 228.43 ± 0.29, 229.33 ± 0.63, and 230.73 ± 0.21 nm (average ± standard deviation, sample number n = 5) for 0, 0.025, 0.050, 0.150, and 0.250 M sucrose water solutions, respectively. As shown in Fig. 3b, there is a clear positive relationship between the refractive index *n* and the SPR wavelength, presenting a possibility for using the SPR sensor in the DUV region. Both water and sucrose have no absorption in the wavelength region used for measurements. It should be emphasized here that the present film exhibited the stable SPR properties; i.e. the SPR wavelengths measured on multiple days were almost the same, leading to the narrow error bars in Fig. 3b; in spite of the natural oxidation of the Al film. Doherty and Davis reported that the growth of naturally formed aluminum oxide films on Al single crystals stopped about 3 nm thickness^30^, which means that the Al film can be used as the stable sensor after the enough oxidation.

**Figure 4:**
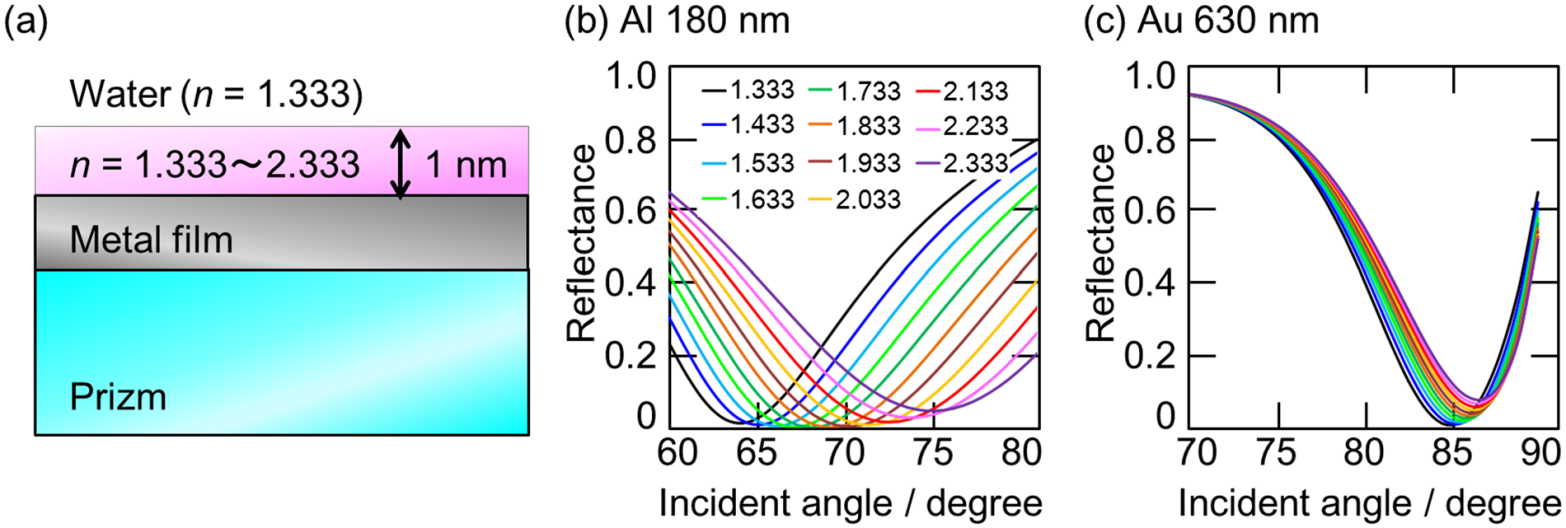
(**a**) A simulation model and (**b**,**c**) calculated incident angle dependence of (**a**) a 15 nm thick Al film on a sapphire prism at 180 nm wavelength and (**b**) a 40 nm thick Au film on a quartz prism at 630 nm wavelength with varying refractive indices from 1.333 to 2.333 in the thickness region of 1 nm on the metal film.

As ascribed in the introduction, the FUV-DUV-SPR sensor are expected to have three advantages compared to the conventional visible-SPR sensors; high sensitivity, material selectivity, and surface measurement accuracy. In order to discuss these advantages in the water solution, simulations based on the model shown in Fig. 4a were done. In the simulation model, the refractive index in only 1 nm thick region on the metal surface was changed from 1.333 to 2.333, and the refractive index on the 1 nm thick film was settled at 1.333 assuming water. The SPR sensor is often used for the detection of antigen-antibody reactions using the metal film coated with antibody molecules^1^, and refractive index changes within the narrowly limited depth from the metal surface are detected in aqueous solutions. Because of these reasons, this model was adopted. A 15 nm thick Al film was put on a sapphire prism as an FUV-DUV sensor, while a 40 nm thick Au film was put on a quartz prism as a visible sensor. These metal film thicknesses were optimized to exhibit deep dips in water (*n* = 1.333). Although the SPR wavelength are mainly discussed in the present study as shown in Figs 1 and 3, the SPR angle is also important factor for evaluating the ability as a sensor. In addition, it is difficult to consider the chromatic dispersion of the refractive index in the FUV and DUV region as described above. Thus, incident angle dependences were calculated based on the model.

As shown in Fig. 4b, in the case of the Al-based FUV-DUV sensor, the SPR angle shifts from 64° to 76° at 180 nm wavelength when the refractive index in the 1 nm thick region on the metal surface changes from 1.333 to 2.333. On the other hand, the SPR angle for the visible sensor shifts only 2° at 630 nm wavelength; from 85° to 87°; which is one-sixth of that for the FUV sensor. Therefore, the Al-based FUV-DUV-SPR sensor is expected to have the surface measurement accuracy. In addition, as mentioned above, materials have larger refractive indices in the FUV and DUV regions than in the visible region, leading to still higher selectivity. When a target material has absorption at the incident wavelength, its refractive index is even higher around the resonant wavelength, resulting in the material selectivity. Many target materials of the SPR sensor such as DNA and antigen proteins have strong absorption in the FUV and DUV regions not in the visible region. Hence, it can be concluded that the FUV-DUV-SPR sensor has the potential as a superior sensor compared to the conventional visible-SPR sensor.

## Conclusions

In summary, the Al-SPR wavelength dependence on the refractive indices of the prisms (quartz and sapphire) and the materials placed on the Al thin film (air, HFIP, water, and sucrose water solutions) were systematically investigated. On placing materials on the Al film, the SPR wavelength shifted to longer wavelengths. When the Al film was evaporated on the quartz prism, the SPR wavelength shifted to the visible region in the presence of water. On the other hand, the SPR wavelength of the Al film on the sapphire prism remained in the DUV region even in water. The advantages of the Al-based FUV-DUV-SPR sensor compared to the Au-based visible-SPR sensor were examined by the simulations assuming that the refractive index in the narrowly limited thick (1 nm) region changes in water, and it was revealed that the SPR angle shift of the Al film at 180 nm wavelength was six times larger than that of the Au film at 630 nm wavelength, which showed a possibility of a novel FUV-DUV-SPR sensor in aqueous solutions with higher sensitivity, material selectivity, and surface measurement accuracy.
